# Health-related quality of life in Parkinson’s disease: a cross-sectional study focusing on non-motor symptoms

**DOI:** 10.1186/s12955-015-0281-x

**Published:** 2015-06-20

**Authors:** Liis Kadastik-Eerme, Marika Rosenthal, Tiiu Paju, Mari Muldmaa, Pille Taba

**Affiliations:** Department of Neurosurgery and Neurology, Tartu University Hospital, L. Puusepa 8, Tartu, 51014 Estonia

**Keywords:** Depression, Health-related quality of life, Impulse control disorders, MDS-UPDRS, Non-motor symptoms, Multiple regression analysis, Parkinson’s disease, PDQ-39

## Abstract

**Background:**

The objective of this study was to investigate factors affecting health-related quality of life (HRQoL) among Estonian persons with Parkinson’s disease (PD).

**Methods:**

268 persons with PD were evaluated using: the Movement Disorder Society Unified Parkinson’s Disease Rating Scale (MDS-UPDRS); the Hoehn and Yahr scale (HY); the Schwab and England Activities of Daily Living scale (SE-ADL); the Beck Depression Inventory (BDI); the Mini Mental State Examination (MMSE); the Parkinson’s Disease Questionnaire (PDQ-39). Additional questions on clinical and socio-demographic variables were asked during a semi-structured interview. Predictors of HRQoL were tested using multiple regression analysis.

**Results:**

The main predictors of low HRQoL were depression and motor and non-motor aspects of daily living. 59.9 % of the variation in the PDQ-39 summary index (SI) score was explained by the predictive variables identified in this study. None of the socio-demographic variables (age, gender, urban/rural living, marital status, living alone/with others, education level) were significant predictors of HRQoL. Prevalence of non-motor Parkinson’s symptoms were high (99.6 %); cognitive impairment, sleep and urinary problems were the most common. All non-motor symptoms correlated significantly with low HRQoL, except the features of impulse control disorders (ICDs).

**Conclusions:**

Depression and motor and non-motor daily living experiences were found to be significant and independent factors of low HRQoL in persons with PD. Depression was the strongest determinant of low HRQoL. Our results highlight the importance of recognition and management of non-motor symptoms, as these features had more impact on patients’ HRQoL than clinically assessed motor symptoms.

## Background

Parkinson’s disease (PD) is one of the most common neurodegenerative diseases worldwide, with a prevalence ranging from 31–201 per 100,000 population [1]. The symptoms of PD encompass motor features such as rigidity, bradykinesia, tremor and postural instability [2], and non-motor symptoms including impairment of olfaction, vision, sleep, salivation, gastric and bowel function, sebaceous gland activity, and mood and cognition [3]. PD diagnosis is based upon the presence of a set of cardinal motor signs; progression is defined by the degree of motor disability; management is primarily directed towards improving motor symptoms [2, 4].

Persons with PD are particularly vulnerable to deterioration of health-related quality of life (HRQoL) resulting from significant motor disability and the burden of non-motor symptoms. Assessment of HRQoL of persons with PD is thus of essential importance. Several factors have been reported to be associated with low HRQoL. Among motor symptoms, the most significant determinants affecting HRQoL are disease severity, motor complications, postural instability and gait disorder [5–7]. Among non-motor symptoms, depression, anxiety, cognitive impairment, fatigue, pain, urinary disturbances and sleep problems were found to be the most significant determinants of HRQoL [5, 8, 9]. Among other factors, a high levodopa equivalent daily dose and comorbidities were found to be prevalent and independent determinants of HRQoL [6]. Parallel to the clinical features, several socio-demographic factors such as level of education and number of people in a household have been described as independent determinants of HRQoL [5, 7].

Most previous studies however have assessed only a limited number of factors in relation to HRQoL. The impact of certain variables (*e.g.* rural living, marital status, education, comorbidities) have been infrequently systematically studied. The main aim of our study therefore was to evaluate the impact of a wide range of socio-demographic and clinical factors upon the HRQoL of a cohort of persons with PD in Estonia. We also focused on the non-motor symptoms identified by the new version of the Movement Disorder Society Unified Parkinson’s Disease Rating Scale (MDS-UPDRS) Part I, which has recently been adopted and validated [10].

## Methods

### Participants

A total of 268 persons with PD were recruited for a community-based study by the Department of Neurology and Neurosurgery of Tartu University Hospital, which is one of two regional hospitals in Estonia. The study was based on a PD cohort derived from an epidemiological study primarily aimed at investigating the epidemiology, clinical characteristics and treatment of PD in Estonia. The current study was conducted in the city and district of Tartu between October 2010 and May 2013. Patient selection was made via evaluation of information from hospital records, neurologists and general practitioners, nursing homes, the local PD Society, and the database of the Estonian Health Insurance Fund. Only patients who fulfilled the Queen Square Brain Bank Criteria [2, 11] were included in the study. As this was an epidemiological study, all patients with a confirmed PD diagnosis were enrolled, and no specific exclusion criteria were set. The study was approved by the Research Ethics Committee of the University of Tartu and all patients provided signed informed consent.

### Materials and procedure

The demographic and social data (age, gender, urban/rural living, marital status, living alone/with others, level of education) and data regarding PD (age at disease onset, disease duration, clinical subtype) and other clinical factors (levodopa equivalent daily dose, duration of levodopa treatment, comorbidities) were recorded using specially developed case report forms. Disease subtypes were based upon the most prevalent symptom during standard neurological examinations: (1) tremor; (2) bradykinesia-hypokinesia; (3) postural instability and gait disorder.

To evaluate HRQoL, PDQ-39—a validated, disease-specific quality of life instrument—was used [12]. The higher the PDQ-39 summary index (SI) score, the lower the perception by patients of their quality of life. An Estonian version of the PDQ-39 has been shown as a reliable instrument [13].

Non-motor PD symptoms were identified using Part I of the MDS-UPDRS. In 2008 the Movement Disorder Society (MDS) adopted a new, validated version of the MDS-UPDRS, which included several significant updates in comparison with the previous version, including new non-motor symptoms of PD [10]. The Estonian version of the MDS-UPDRS was translated, validated and officially approved in 2011 by the MDS Translation Program for non-English official versions. The MDS-UPDRS Part IA contains questions regarding a number of neuropsychiatric symptoms and is completed by the interviewer; Part IB consists of questions on non-motor symptoms and is answered by the patient or caregiver. Other parts of the MDS-UPDRS were used: Part II regards the motor experiences of daily living as assessed by the patient or caregiver; Part III assesses motor symptoms based on an objective neurological examination; Part IV regards motor complications and is completed by the interviewer.

The clinical stage of a patient’s PD was assessed using the Hoehn and Yahr scale (HY) [14] and their level of disability by the Schwab and England Activities of Daily Living Scale (SE-ADL) [15]. The HY scale ranges from 1 (unilateral involvement of the body) to 5 (wheelchair bound or bedridden). The SE-ADL scale ranges from 0 % (completely dependent and bedridden) to 100 % (completely independent). For assessment of depression the Beck Depression Inventory (BDI) was used [16], whereby a score of ≥14 is indicative of depression [17]. Cognitive impairment was measured using the Mini Mental State Examination (MMSE) [18], whereby a score of ≤24 is evidence of cognitive impairment.

### Statistical analysis

PDQ-39 SI measures of HRQoL were calculated according to its scoring algorithm [12, 19]. Prevalence of non-motor symptoms was based on scores ≥1, which denoted the presence of a symptom. Student’s *t*-test and z-test were applied for comparing socio-demographic and clinical outcomes between men and women. The non-parametric Mann–Whitney and Kruskal-Wallis tests were used to compare PDQ-39 SI scores between the groups. Spearman’s rank correlation coefficients were calculated to assess associations between variables. Correlation coefficients were interpreted as very weak (*r* = 0–0.19), weak (*r* = 0.20–0.39), moderate (*r* = 0.40–0.59), strong (*r* = 0.60–0.79) or very strong (*r* = 0.80–1.00).

A multiple linear regression analysis—based on a backward elimination approach—was conducted to determine the factors that contribute to HRQoL in persons with PD. PDQ-39 SI was used as a dependent variable. The R^2^ statistic was used to determine the proportion of variance explained by the predictors. The level of statistical significance was set at p < 0.05. Statistical analysis was performed using STATA version 12.0 (StataCorp LP, College Station, TX, USA) and SPSS version 20.0 (IBM Corporation, Armonk, NY, USA).

## Results

### Patient characteristics

**Fig. 1:**
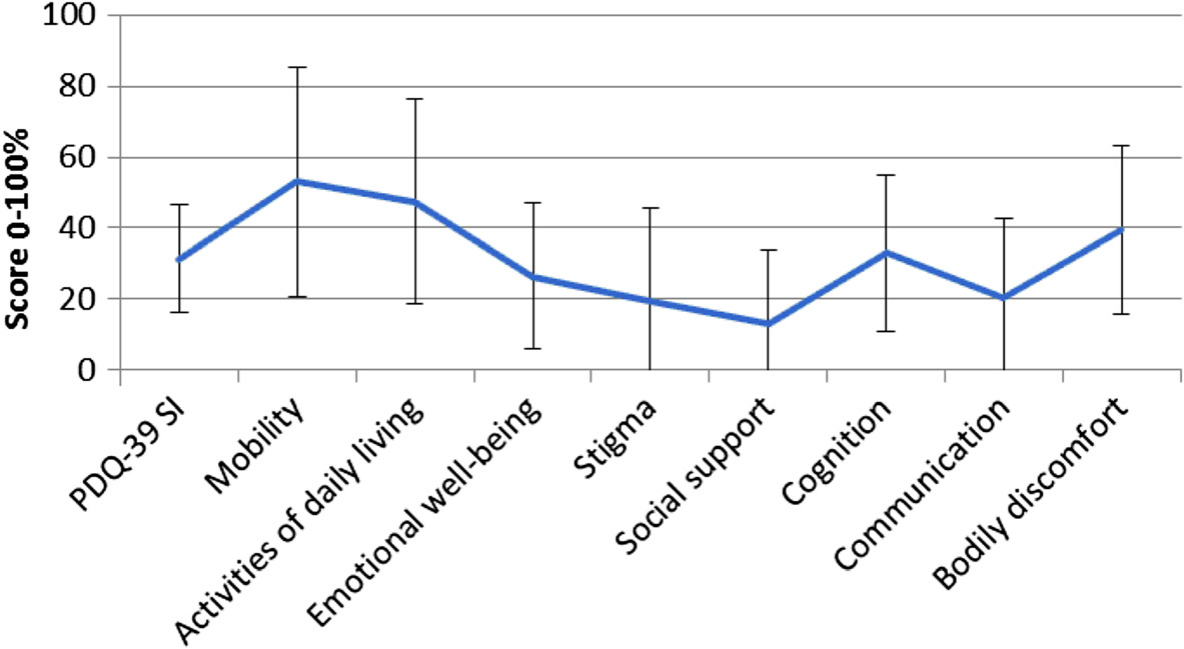
Mean values of PDQ-39 domains. HRQoL was assessed by using the PDQ-39. PDQ-39 is composed of 39 items grouped in 8 subscales: (1) mobility, (2) activities of daily living, (3) emotional well-being, (4) stigma, (5) social support, (6) cognition, (7) communication, and bodily discomfort. The PDQ-39 SI is an overall score calculated from these domains, with 0 indicating the best HRQoL and 100 the worst

Demographic and clinical characteristics of the study population are given in Table 1. Men were slightly younger than women (72.1 and 75.6 years respectively, p = 0.0014), which reflects the shorter life expectancy of men in Estonia. No significant differences in terms of PD onset age, disease duration, HY stage, SE-ADL stage or MMSE performance were found between men and women. The mean BDI depression score was significantly higher in women compared to men (17.1 and 12.3 respectively, p = 0.0001). A significantly higher rate of men compared to women were: married (71.4 % and 32.5 % respectively, p < 0.0001); lived with their spouse and or children (89.5 % and 60.1 % respectively, p < 0.0001). There were more widows among female than male patients (48.5 % and 17.1 % respectively, p = 0.02). Women had a higher mean number of comorbidities compared to men (2.23 and 1.86 respectively, p = 0.0124), including a significantly higher rate of cardiovascular diseases (99.4 % and 89.6 % respectively, p < 0.0001). Mean scores of PDQ-39 domains are presented in Fig. 1. In persons with PD, mobility (mean score 53.1) was the most negative HRQoL domain and social support (mean score 13.17) the least negative domain.

**Table 1:** Characteristics of the patients

Variable	Value	Range
Age ^a^	74.2 yr (±8.8)	47-96
Disease onset age ^a^	66.8 yr (±10.1)	35-88
Duration of disease ^a^	7.6 yr (±5.9)	1-35
HY ≥3, n (%)	164 (62.6 %)	
SE-ADL ≤75 %, n (%)	139 (53.3 %)	
MMSE ≥25, n (%)	197 (76.7 %)	
BDI ≥14, n (%)	134 (52.1 %)	
MDS-UPDRS		
MDS-UPDRS Part I	12.52 (6.79)	0-38
MDS-UPDRS Part II	18.2 (8.4)	1-48
MDS-UPDRS Part III	46.3 (18.3)	10-106
MDS-UPDRS Part IV	1.3 (3.4)	0-18
Clinical subtypes		
Tremor dominant	118 (44 %)	
Hypokinetic-rigid dominant	77 (28.7 %)	
Postural instability and gait disturbance dominant	73 (27.3 %)	
Levodopa equivalent dose per day ^a^	427.5 mg (±231.6)	100-1200
Duration of levodopa treatment ^a^	3.9 yr (±5.03)	0.1-23
Marital status, n (%)		
Single	20 (7.4 %)	
Married	128 (47.8 %)	
Divorced	23 (8.6 %)	
Widowed	97 (36.2 %)	
Living status, n (%)		
With a spouse	137 (51,1 %)	
With children	55 (20,5 %)	
Alone	66 (24.6 %)	
Nursery home	10 (3.8 %)	
Education, n (%)		
Primary		
Secondary	104 (38.8 %)	
Higher	95 (35.5 %)	
	69 (25.7 %)	

^a^ Mean (standard deviation)

*Abbreviations*: HY, Hoehn and Yahr stage; SE-ADL, Schwab and England Activities of Daily Living Scale; MMSE, Mini Mental State Examination; BDI, Beck Depression Inventory; MDS-UPDRS, Movement Disorders Society Unified Parkinson’s Disease Rating Scale

### Prevalence of non-motor symptoms

Among the 268 patients screened, 99.6 % complained of at least one non-motor symptom. There were a mean number of 6.7 non-motor symptoms (SD 2.5) per patient of the 13 symptoms included in the MDS-UPDRS Part I. The most frequent non-motor symptoms were cognitive impairment, night time sleep disorders, bladder disorders, fatigue, pain, daytime sleepiness and depression. Hallucinations and impulsive compulsive disorders (ICDs) were the most infrequently reported non-motor symptoms. We did not find a statistically significant difference between mean BDI scores for persons with PD and with ICDs, and persons with PD and without ICDs (mean score 19.1 and 15.1, respectively, p = 0.254). Prevalence of non-motor symptoms is given in Table 2.

**Table 2:** Prevalence of non-motor symptoms

Non-motor symptoms	N	%
Cognitive impairment	199	74.3 %
Nighttime sleep problems	192	71.6 %
Urinary problems	192	71.6 %
Fatigue	184	68.7 %
Pain	172	64.2 %
Daytime sleepiness	166	61.9 %
Depressed mood	163	60.8 %
Constipation problems	156	58.2 %
Anxious mood	151	56.3 %
Lightheadedness on standing	140	52.2 %
Apathy	122	45.7 %
Hallucinations and psychosis	37	13.8 %
ICDs	21	7.8 %

*Abbreviations*: ICDs, impulse control disorders

### PDQ-39 and socio-demographic factors affecting HRQoL

The results of univariate analysis of socio- demographic and clinical parameters and mean scores of PDQ-39 SI in persons with PD are shown in Table 3. Emotional well-being and bodily discomfort affected women significantly more than men. Being over 65 years old was associated with lower mobility: being younger was associated with higher stigmatisation. Marital status was not significantly associated with PDQ-39 scores. Emotional well-being was significantly related to living status, *i.e.* patients living alone had lower emotional well-being compared to patients living with their spouse and/or children. Education did not significantly influence overall HRQoL. People who had undergone no more than primary level education were significantly more affected in the domain of mobility.

**Table 3:** PDQ-39 associations with demographic and clinical features

Variable	PDQ-39 SI^a^	p value
Gender		0.066
Male	29.8 (15.5)	
Female	32.7 (15.1)	
Living area		0.192
Urban	32.5 (15.3)	
Rural	29.6 (15.1)	
Marital status		0.770
Single	32.7 (17.4)	
Married	30.8 (15.3)	
Divorced	34.2 (13.1)	
Widowed	31.7 (15.4)	
Living status		0.638
With a spouse	30.6 (15.4)	
With children	31.5 (17.1)	
Alone	33.5 (13.5)	
Nursery home	32.7 (15.3)	
Education		0.339
Primary	33.6 (13.6)	
Secondary	30.4 (15.1)	
Higher	30.1 (17.6)	
Clinical subtypes		0.0017; 1vs 3
Tremor	28.6 (14.9)	
Hypokinetic-rigid	30.5 (14.4)	
Postural instability and gait disturbance	37.3 (15.3)	
Disease duration		0.00010; 1 vs 3
≤5 yr	26.5 (13.0)	
6-10 yr	32.1 (16.1)	
>10 yr	38.8 (14.8)	
HY		<0.0001
≤2.5	24.2 (13.9)	
≥3	35.7 (14.6)	
SE-ADL		<0.0001
≥80	25.0 (14.4)	
≤75	37.0 (14.0)	
MMSE		0.0037
≥25	29.9 (14.9)	
≤24	35.9 (14.6)	
BDI		<0.0001
<14	23.0 (11.9)	
≥14	39.2 (14.1)	

^a^ Mean (standard deviation)

*Abbrevations*: HY, Hoehn and Yahr stage; SE-ADL, Schwab and England Activities of Daily Living Scale; MMSE, Mini Mental State Examination; BDI, Beck Depression Inventory

### PDQ-39 and clinical factors affecting HRQoL

Overall HRQoL was significantly lower in patients with depression, which had an impact on all the PDQ-39 domains. Longer disease duration, a HY stage ≥3 and SE-ADL ≤75 % were associated significantly with lower overall HRQoL and affected most of the PDQ-39 domains. Patients with cognitive impairment had lower overall HRQoL and were significantly more adversely affected in the domains of mobility, activities of daily living and communication.

By clinical PD subtype, the group with postural instability and gait disturbance had the lowest overall HRQoL (compared to the tremor and hypokinetic-rigid clinical subtypes). Out of PDQ-39 domains, mobility and activities of daily living had the lowest outcome.

### Correlation analysis between PDQ-39 SI and socio-demographic and clinical factors

PDQ-39 SI correlated significantly with higher MDS-UPDRS Part II, BDI and MDS-UPDRS Part IB scores. All non-motor symptoms—except ICDs—correlated significantly with PDQ-39 SI. All correlations between PDQ-39 and the other characteristics of PD are shown in Table 4.

**Table 4:** Spearman’s correlation analysis of clinical variables and PDQ-39 SI scores

Variable	Coefficient	p value
Disease duration	0.30	<0.0001
Clinical subtypes		
Tremor dominant	-0.17	0.006
Hypokinetic-rigid dominant	-0.05	0.449
Postural instability and gait disturbance dominant	0.23	<0.0001
HY	0.51	<0.0001
SE-ADL	-0.51	<0.0001
MMSE	-0.25	0.0001
BDI	0.63	<0.0001
Daily dose of levodopa	0.32	<0.0001
Duration of levodopa treatment	0.23	0.0001
Number of comorbidities	0.07	0.5041
MDS-UPDRS Part I – Non-motor experiences of daily living	0.62	<0.0001
MDS-UPDRS Part IA	0.51	<0.0001
MDS-UPDRS Part IB	0.61	<0.0001
Cognitive impairment	0.38	<0.0001
Hallucinacions and psychosis	0.24	0.0001
Depressed mood	0.35	<0.0001
Anxious mood	0.29	<0.0001
Apathy	0.33	<0.0001
ICDs	0.07	0.2490
Nighttime sleep problems	0.29	<0.0001
Daytime sleepiness	0.35	<0.0001
Pain and other sensations	0.38	<0.0001
Urinary problems	0.30	<0.0001
Constipation problems	0.13	0.0281
Lightheadedness on standing	0.34	<0.0001
Fatigue	0.44	<0.0001
MDS-UPDRS Part II – Motor experiences of daily living	0.65	<0.0001
MDS-UPDRS Part III – Motor examination	0.46	<0.0001
MDS-UPDRS Part IV – Motor complications	0.22	0.0003

*Abbrevations: PDQ*-39, The Parkinson’s Disease Questionnaire; SI, summary index score; HY, Hoehn and Yahr stage; SE-ADL, Schwab and England Activities of Daily Living Scale; MMSE, Mini Mental State Examination; BDI, Beck Depression Inventory; MDS-UPDRS, Movement Disorders Society Unified Parkinson’s Disease Rating Scale; ICDs, Impulse control disorders

### Multiple linear regression model of PDQ-39 SI

Potential predictors were determined based on the previous correlation analysis. Duration of disease, HY, SE-ADL, MMSE, BDI, MDS-UPDRS Parts IA, IB, II-IV, duration of levodopa treatment, daily dose of levodopa, postural instability and gait disorder or tremor dominated disease types were identified as independent variables using univariate regression analysis. Multiple linear regression analysis was then performed on all the independent variables found during the previous univariate regression analysis and common epidemiological variables, *i.e.* age and gender. The final model (Table 5) explained 59.9 % (adjusted R^2^, p < 0.0001) of the variance of PDQ-39 SI, with BDI, MDS-UPDRS II, MDS-UPDRS Part IB significant predictors and depression the most significant predictor of HRQoL.

**Table 5:** Predictors of HRQoL in stepwise multiple regression analysis

Model	Unstandardized coefficients		p value
	B	SE	
BDI	0.734	0.089	0.0001
MDS-UPDRS Part IB	0.603	0.201	0.003
MDS-UPDRS Part II	0.546	0.127	0.0001
MDS-UPDRS Part III	0.067	0.050	0.182
Age	-0.124	0.077	0.108
Duration of the disease	0.251	0.184	0.175
Duration of levodopa treatment	-0.339	0.211	0.109
	Multiple	R^2^ = 0.611	
	Adjusted	R^2^ = 0.599	

HRQoL, health-related quality of life; SE, standard error; BDI, Beck depression inventory; MDS-UPDRS, Movement Disorder Society Unified Parkinson’s Disease Rating Scale

## Discussion

The purpose of this study was to investigate factors that may contribute to low HRQoL in persons with PD. We found that the main clinical determinants of low HRQoL in persons with PD were depression and the high burdens of motor and non-motor aspects of daily living. None of the investigated socio-demographic variables significantly associated with HRQoL.

Depression has an average prevalence of about 40 % among persons with PD [20] and is a recognized predictor of low HRQoL. As previous studies [5, 8], we found depression to be one of the most significant determinant of HRQoL. A study by Leentjens *et al.* [21] demonstrated that the risk factors in the general population for depression—such as older age, female gender, somatic comorbidities, and personal and family history of depression—also predict depression in persons with PD. In our sample the prevalence of depression in female patients was significantly higher than in male patients (58 % and 43 % respectively, p = 0.0189). In addition to BDI, depression was more often reported by women according to Part IA of the MDS-UPDRS. Studies examining gender differences regarding prevalence of depression have yielded inconsistent results. While van der Hoek *et al.* [22] found no difference in the prevalence of depression in male and female persons with PD, Solla *et al.* [23] found prevalence of depression significantly higher in female persons with PD. The reason why women are more disposed to depression might partly be explained by the greater exposure of competing risk factors, as suggested by Sonnenberg *et al.* [24] who demonstrated this association in a study on gender differences for depression in the elderly of a general population: controlling for age, comorbid somatic disease, and a number of other risk factors reduced the relative risk of depression in women by more than a half. The female patients in our study were slightly older than the male patients, were more often widowed and had a higher mean number of comorbidities. Men in our cohort were significantly more often married and lived together with their wife and or children. Based on the studies by Sonnenberg *et al.*, it could be assumed that controlling for several factors associated with social and health status might reduce the relative risk of depression in female persons with PD.

In recent years there has been increasing evidence suggesting that the impact of non-motor symptoms on HRQoL is more important than the impact of motor features [8, 9]. Similar to previous studies, our study revealed that HRQoL was significantly lower with higher loads of non-motor symptoms. All non-motor symptoms correlated significantly with PDQ-39 SI, except the features of ICDs: a heterogeneous group of pathological behaviours associated with dopamine replacement treatment that include pathological gambling, compulsive sexual behaviour, compulsive shopping, and binge eating, together with pounding and the addiction-like compulsive use of dopamine replacement therapy [25]. A case study by Voon *et al.* indicated that patients with ICDs have more depressive symptoms [26]. We did not find a statistically significant difference between mean BDI scores for persons with PD and with ICDs (based on positive item 1.6 in MDS-UPDRS Part IA), and persons with PD and without ICDs. Reducing the dosage or discontinuing the administration of dopaminergic agonists or substituting another drug from this group is a frequently effective therapeutic measure [25, 27]. Only a few studies on the impact of ICDs on HRQoL have been published and some found controversial results, *i.e.* ICDs as a predictor of lower HRQoL [28, 29], but in other studies [30] ICDs did not affect HRQoL. The current study did not reveal a negative impact of ICDs on quality of life. Our finding is supported by a study by Ondo, where only 18 % of patients with increased impulsivity felt that the change was deleterious [30]. Our daily clinical practice suggests that ICDs cause considerable distress to patients’ families or caregivers, but the patients themselves are not that annoyed by these behaviours. Out of the 268 patients screened in our study, 7.8 % (n = 21) complained of ICDs. It could be assumed that at least one reason why ICDs did not correlate significantly with HRQoL is that the pathological behaviours were relatively mild. About half (52 %) of these patients reported only slight problems with behavioural disturbances and it could be that the problems did not yet affect their social and occupational functioning.

Dependency in activities of daily living as assessed using the SE-ADL scale and disability in the performance of daily living experiences as measured by the previous version of the UPDRS Part II, have been found to be significant contributors to HRQoL [5]. In a recently published study by Rodriguez-Blazquez *et al.* [31], who used the MDS-UPDRS Part II, a significant negative association between the more advanced disability and HRQoL occurred. The current study demonstrated that disability evaluated by SE-ADL and MDS-UPDRS Part II significantly contributed to lower HRQoL. However, motor symptoms (MDS-UPDRS Part III) were not an independent predictor of HRQoL. Even though for antiparkinsonian treatment Part III with motor assessments is of high importance, it can be seen that for HRQoL, the self-assessment of non-motor symptoms and ability to perform daily activities have more impact.

An axial impairment has been shown to be associated with reduced HRQoL in persons with PD [6]. We found that patients with postural instability and gait disorder dominance had significantly lower overall HRQoL compared to patients with tremor dominance or hypokinetic-rigid dominance; however, in multiple regression analysis it did not appear to be an independent predictor of HRQoL. Neither was HY stage found to be an independent predictor, though patients with more severe PD (HY ≥3) had lower HRQoL than patients with milder PD. The results of the multiple regression analysis were concordant, as higher HY stages reflected an axial involvement with balance and gate impairments. Persons with PD in general, but particularly in patients with the postural instability and gait disorder, are likely to become less able to move around inside their homes and out in the community. They also become more prone to falls as the disease progresses. Regular physical exercise is associated with higher HRQoL, mobility, physical function, slowing the progression of the disease, lower caregiver burden, and less cognitive decline [32]. In view of this, patients with the postural instability and gait disorder may benefit most from education on using rehabilitation activities (*e.g.* physical exercise, physiotherapy) and assistive devices (*e.g.* reaching aids). Therefore, in addition to pharmacological treatment, the management of PD should include rehabilitative care, which helps to maintain patients’ ability to participate in daily living activities and avoid the decline of HRQoL.

In our study socio-demographic characteristics including age, gender, urban/rural living, level of education, marital status and living alone/with others, did not significantly affect overall HRQoL. However, we found several associations between these factors and domains of PDQ-39. Women received lower scores for emotional well-being and bodily discomfort, which is in accordance with the study by Carod-Artal *et al.* [5]. As regards to patients’ living status, those who lived alone received lower scores for emotional well-being compared with patients who lived with others. Another study by Winter *et al.* showed that patients living alone had worse HRQoL than patients living with somebody [7]. Our results did not reveal any statistically significant difference in HRQoL among patients with different educational backgrounds, whereas greater number of years in education was found to be associated with higher HRQoL in a study by Carod-Artal *et al.* [5]. Klepac *et al.* [33] found that rural patients had lower overall HRQoL, with most of the domains of PDQ-39 affected. In contrast, the results of our study suggest that living area is not associated with overall HRQoL. Moreover, our results showed that patients living in rural areas had significantly less stigmatization and better social support than patients living in urban areas.

The study has some methodological limitations that ought to be recognized and taken into account when interpreting the findings. The analysis was based on clinical data collected at a single point in time; therefore, any pattern of progression of the disease could not be estimated. Also, we cannot exclude the possibility that some of the variables, such as gender (female patients outnumbered male patients) or stage of disease (62.6 % of patients had HY ≥3) could have influenced the results found.

The strength of the study was the relatively large sample of persons with PD, which included institutionalised and severely ill patients, and thus was representative of the PD population as a whole. The study participants were also evaluated with a wide range of clinimetric properties that covered motor, non-motor, functional, cognitive and emotional aspects.

## Conclusion

The results of our study suggest that both non-motor and motor aspects affect the daily living and HRQoL of persons with PD. Our results are consistent with other studies that have suggested depression to be the strongest predictor of HRQoL in persons with PD and therefore depression should be recognized early and treated appropriately. Attention should also be paid to other common non-motor symptoms that may require a multidisciplinary approach, such as cognitive impairment, sleep problems, urinary problems and fatigue. A novel observation of this study was that ICDs were the only non-motor symptom not associated with lower HRQoL. Further research in this setting is planned to explore the role of behavioural disturbances in HRQoL among persons with PD.

## Abbreviations

BDI: Beck Depression Inventory
HRQoL: Health-Related Quality of Life
HY: Hoehn and Yahr scale
ICDs: Impulse Control Disorders
MDS-UPDRS: Movement Disorders Society Unified Parkinson’s Disease Rating Scale
MMSE: Mini Mental State Examination
PD: Parkinson’s Disease
PDQ-39: Parkinson’s Disease Quality of Life Questionnaire
SE-ADL: Schwab and England Activities of Daily Living scale
SI: Summary Index
